# Amorphized length and variability in phase-change memory line cells

**DOI:** 10.3762/bjnano.11.147

**Published:** 2020-10-29

**Authors:** Nafisa Noor, Sadid Muneer, Raihan Sayeed Khan, Anna Gorbenko, Helena Silva

**Affiliations:** 1 Department of Electrical and Computer Engineering at North South University, Dhaka, Bangladesh; 2 Department of Electrical and Electronic Engineering at United International University, Dhaka, Bangladesh; 3 Department of Electrical and Computer Engineering at University of Connecticut, Storrs, CT-06269, USA

**Keywords:** amorphous materials, drift, electrical breakdown, electrical resistivity, phase-change memory, pulse measurement, stochastic processes, threshold switching

## Abstract

The dimensions of amorphized regions in phase-change memory cells are critical parameters to design devices for different applications. However, these dimensions are difficult to be determined by direct imaging. In this work, the length of amorphized regions in multiple identical Ge_2_Sb_2_Te_5_ (GST) line cells was extracted from electrical measurements. After each cell was programmed to an amorphous state, a sequence of increasing-amplitude post-reset voltage pulses separated by low-amplitude read DC sweeps was applied. When a post-reset voltage pulse with sufficient amplitude was applied to a given cell, the measured current and the post-pulse resistance increased drastically, indicating that the cell re-amorphized after threshold switching, melting, and quenching. The amorphized length was calculated using the measured voltage at which the threshold switching occurred and the expected drifted threshold field at that time. The measured threshold voltage values and, hence, the extracted amorphized length, generally increase linearly with the programmed resistance levels. However, significant variability arises from the intrinsically unique crystallization and amorphization processes in these devices. For example, cells programmed to an amorphous resistance of approx. 50 MΩ show threshold voltage values of 5.5–7.5 V, corresponding to amorphized length values of 290–395 nm. This unpredictable programming feature in phase-change memory devices can be utilized in hardware security applications.

## Introduction

Phase-change memory (PCM) is an emerging non-volatile memory technology with high endurance, high speed, and good scalability. PCM relies on the change in phase of a nanoscale volume of a chalcogenide material sandwiched between two electrodes. The phase of the material can be switched between the high-resistivity state (amorphous or reset) and the low-resistivity (crystalline or set) state by appropriate electrical pulses. The amorphization (or reset) process in PCM is achieved with a short and abrupt electrical pulse, which melt-quenches the active region [1]. PCM nanodevices exhibit significant cell-to-cell and cycle-to-cycle programming variability due to the intrinsic randomness in the crystallization and amorphization processes, in addition to any variation in the fabrication process. This probabilistic programming feature in PCM has been recently considered for several applications in hardware security, such as physical unclonable functions or true random number generators [2–7]. It is critical to characterize the physical factors that contribute to the observed variability for conclusive understanding and proper utilization of this feature for these hardware security primitives. An essential physical parameter that contributes to the programming variability is the random location and dimensions of different phases formed in the cell. The amorphous and crystalline regions in PCM devices can be distinguished by transmission electron microscopy (TEM) imaging [8]. However, this is a difficult and time-consuming process, and the sample preparation and imaging processes themselves may disturb the state of the material [9–10]. Hence, TEM becomes impractical for variability analysis using a large number of devices.

**Figure 1:**
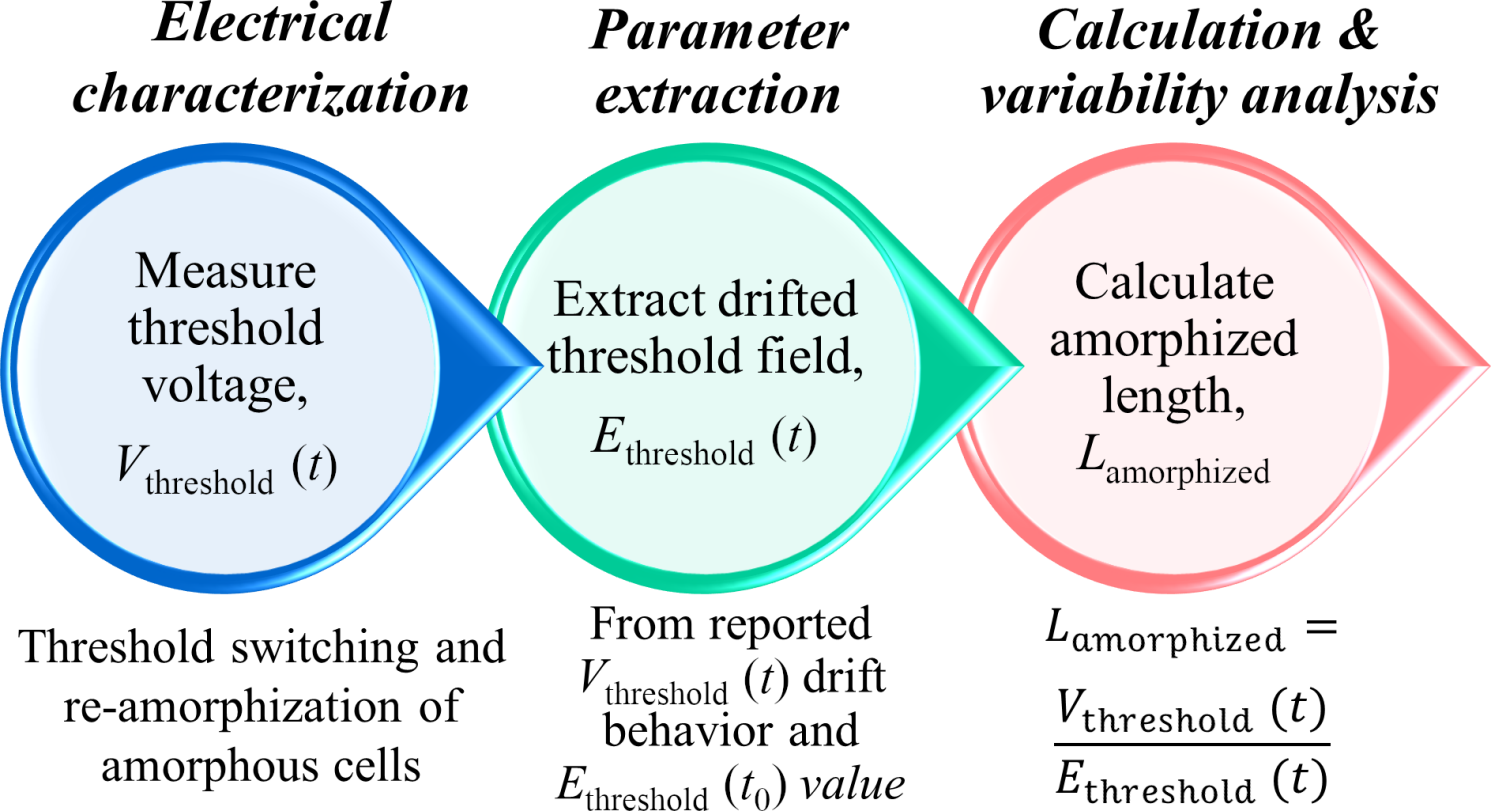
Schematic of the procedure used in this work for extraction of amorphized length in phase-change memory line cells.

In this study, the length values of the amorphized regions are calculated from electrical measurements on 25 Ge_2_Sb_2_Te_5_ (GST-225) PCM line cells of very similar dimensions. The crystalline PCM line cells were first amorphized with one or more reset pulses. Then, a sequence of post-reset pulses was applied with gradually increasing amplitudes. The cells were read after each pulse. When the applied post-reset pulse amplitude was high enough, a significant increase in the measured current and post-pulse cell resistance was observed. This indicated a re-amorphization after threshold switching, melting, and subsequent quenching, as inferred from the measurements and circuit simulation results obtained from the “simulation program with integrated circuit emphasis” (SPICE). The measured threshold voltage, and the drifted threshold field at that time are used to extract the amorphized length (Figure 1).

### A literature review of threshold switching in PCM

Threshold switching is a sudden and reversible physical process in amorphous chalcogenides, which is accompanied by a sharp decrease in resistance. Various models were presented in the 1970s to explain the threshold switching process. Some of these models indicated pure thermal effects [11], whereas some referred to pure electrical effects [12]. Others pointed to a combination of both [13]. The argument was settled for decades with the dominance of the electrical model. However, it was re-ignited in the last decade by the difficulties in understanding threshold switching in PCM cells at the nanoscale. Among several new explanations based on electrical [14–18] and thermally assisted [19] effects, the electrical models are again prevailing, even though the specific mechanisms are expected to depend on the device geometry and dimensions [19].

According to the current understanding, threshold switching is initiated by an electrical breakdown due to impact ionization, a purely electrical effect. The following sharp increase in current is ascribed to thermal runaway (i.e., a decrease in resistivity with increasing temperature that leads to further heating [17]). In this work, we use this model to interpret our measurement results.

Krebs et al. measured a threshold field of 56 V/µm for as-deposited GST amorphous bridge cells by measuring the threshold voltage values of various cells of known dimensions. They concluded that the threshold field is a material-dependent physical parameter, whereas the specific threshold voltage depends on the device geometry [17]. As-deposited amorphous cells of known dimensions were used in the measurements, instead of melt-quenched cells, to avoid the difficulty in determining the dimensions of the amorphized regions. However, the drift time since deposition, which is a critical parameter, is difficult to be extracted. Since the threshold voltage, *V*_threshold_(*t*), drifts upward in time [20], an effective threshold field, *E*_threshold_(*t*), is also expected to drift similarly, while the amorphized length, *L*_amorphized_, is expected to remain the same over time, despite structural relaxation [21] or other physical processes that may occur in the amorphous volume below the glass transition. The relation between *V*_threshold_(*t*), *E*_threshold_(*t*), and *L*_amorphized_ is given by:

[1] $$E_{\text{threshold}}(t)=\frac{V_{\text{threshold}}(t)}{L_{\text{amorphized}}}.$$

Jeyasingh et al. measured a threshold field of 14 V/µm in melt-quenched GST devices [22]. Based on an elapsed time after amorphization of 100 μs, *t*_0_*,* (from another relevant work of the same group [23]) and the threshold field at *t*_0_, *E*_threshold_(*t*_0_), it is possible to extract the threshold field, *E*_threshold_(*t*), at the elapsed time between amorphization and the time, *t*, when *V*_threshold_(*t*) was measured, using the logarithmic trend of threshold voltage drift and the room-temperature threshold voltage drift coefficient, γ = 0.02, reported by Karpov et al. [20]:

[2] $$\frac{V_{\text{threshold}}(t)-V_{\text{threshold}}(t_{\text{0}})}{V_{\text{threshold}}(t_{\text{0}})}=\gamma \ln\left(\frac{t}{t_{\text{0}}}\right),$$

[3] $$\frac{E_{\text{threshold}}(t)-E_{\text{threshold}}(t_{\text{0}})}{E_{\text{threshold}}(t_{\text{0}})}=\gamma \ln\left(\frac{t}{t_{\text{0}}}\right).$$

In this work, we have used a value of *E*_threshold_(*t*_0_) = 14 V/µm, calculated by Jeyasingh et al. at *t*_0_ ≈ 100 μs after amorphization for melt-quenched amorphous line cells, to obtain *E*_threshold_(*t*) at different measured time values, *t* on different cells programmed to a similar amorphous resistance. Equation 3 and γ = 0.02 were used to calculate *L*_amorphized_ and the associated variability. It is important to note that the term “electrical breakdown” here refers to the reversible threshold-switching process and not to the permanent and detrimental electrical breakdown failure that occurs in any dielectric material.

## Experimental

**Figure 2:**
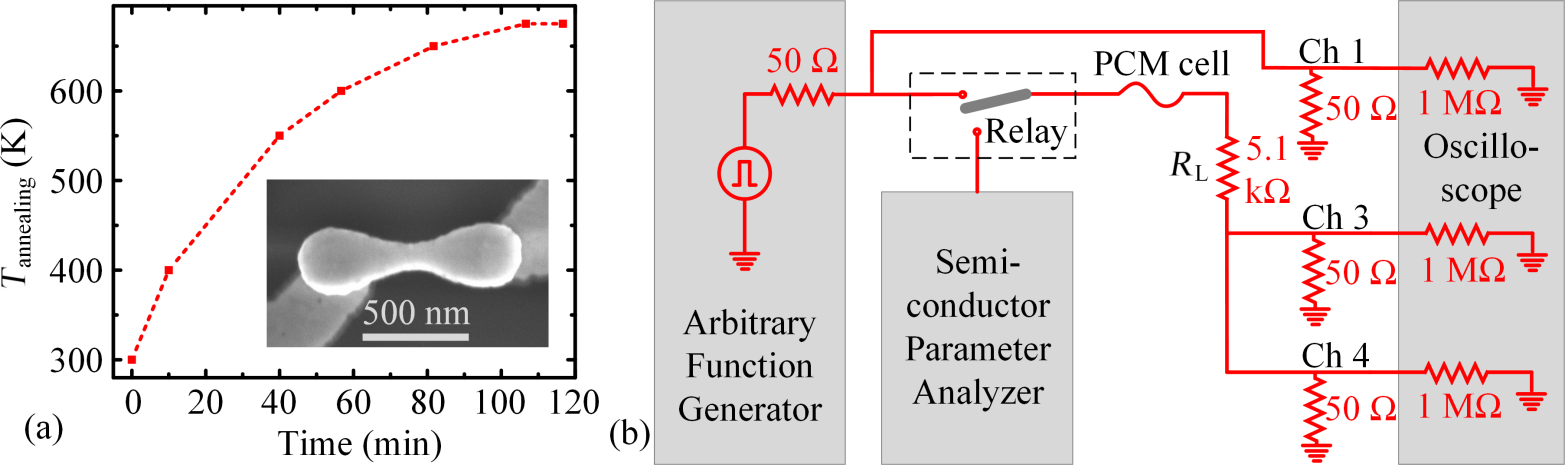
(a) Temperature profile used for annealing the as-fabricated amorphous devices to the crystalline phase, with a constant temperature of 675 K for the last 10 min. Inset shows the SEM image of an untested annealed GST line cell, with metal-to-metal *L*_GST_ ≈ 470 nm, *W*_GST_ ≈ 130 nm, and *t*_GST_ ≈ 50 nm. (b) Electrical measurement setup.

The patterned GST-225 line cells used for this study were deposited on silicon dioxide (SiO_2_), had bottom metal contact pads (tungsten with Ti/TiN liner), and were capped by silicon nitride [24]. All cells were approx. 130 nm in width, *W*_GST_, approx. 470 nm in length between the metal contacts, *L*_GST_, and approx. 50 nm in thickness, *t*_GST_ (SEM image in inset of Figure 2a). The as-fabricated cells were annealed in a Janis ST-500-UHT probe station at a pressure of approx. 1 mTorr at a temperature of 675 K for 10 min to reach the crystalline phase (annealing profile shown in Figure 2a).

The electrical measurements were performed at room temperature. Electrical pulses, generated by an arbitrary function generator (Tektronix AFG 3102), were applied to the cells; a series load resistor of 5.1 kΩ was used to limit the current. The experimental circuit schematic is shown in Figure 2b. The experimental circuit was terminated through a digital oscilloscope (Tektronix DPO 4104) using two termination resistors of 50 Ω connected to the channels 3 and 4. The applied voltage was monitored with the channel 1 of the oscilloscope and the voltage across the parallel combination of two 50 Ω resistors was monitored with channels 3 and 4 to extract the current through the cell. A semiconductor parameter analyzer (Agilent 4156C) was used for low-voltage read operations with a DC voltage sweep between −0.1 and +0.1 V for all resistance levels. A relay circuit, controlled by an Arduino Mega 2560 card, was used to switch between the reading and programming sequences of the measurements.

Twenty-five identical crystalline line cells with similar initial crystalline resistance values (*R*_crystalline_) were amorphized to a similar programmed resistance level (*R*_programmed_) of approx. 10^7^ Ω with a comparatively narrow distribution, so that the variations in the initial *R*_programmed_ value do not greatly affect the extraction of *L*_amorphized_. Table 1 shows the statistical distributions of the *R*_crystalline_ and *R*_programmed_ values for the 25 measured GST cells.

**Table 1:** Statistical distribution of cell resistance values. Minimum, maximum, and median of the crystalline and programmed resistance values (*R*_crystalline_ and *R*_programmed_) of the 25 measured GST cells (*L*_GST_ ≈ 470 nm, *W*_GST_ ≈ 130 nm, and *t*_GST_ ≈ 50 nm).

Cell resistance	Minimum resistance	Maximum resistance	Median

*R*_crystalline_	267 Ω	3162 Ω	522 Ω
*R*_programmed_	8 MΩ	65 MΩ	28 MΩ

**Figure 3:**
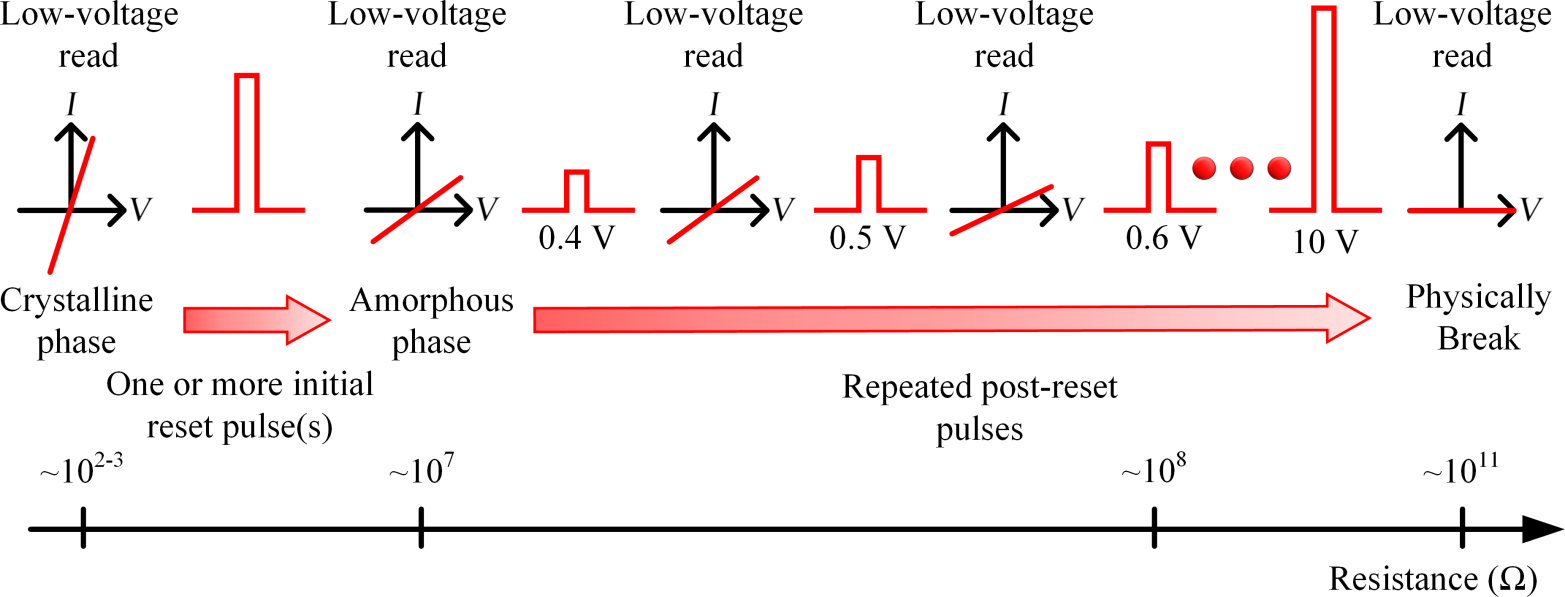
Schematic of the measurement sequence. One or more pulses were applied to initially reset the crystalline cells. Then, a sequence of pulses with increasing amplitude (from 0.4 to 10 V in 0.1 V increment) were applied until further re-amorphization. Eventually, physical breaking occurred with loss of electrical connection. Low-voltage read operations were performed before and after each applied pulse.

Due to intrinsic programming variability and process variations observed from cell to cell, the number and the amplitude of the applied voltage pulses required to reach *R*_programmed_ values of approx. 10^7^ Ω varied [7]. The number of pulses varied between one and ten and the amplitude varied between 2.0 and 2.5 V. These reset pulses were applied with gradually increasing amplitude, in increments of 0.1 V. All amorphization voltage pulses were rectangular with a duration, *t*_duration_, of approx. 200 ns and rise and fall time values, *t*_rise_ *= t*_fall_, of approx. 25 ns. Once the cells reached this initial reset condition, a sequence of rectangular “post-reset pulses”, with gradually increasing amplitudes (0.4 to 10.0 V with 0.1 V increment), and the same *t*_duration_, *t*_rise_, and *t*_fall_ values as used in the initial reset step were applied. Low-voltage DC sweep read operations were performed before and after each applied pulse. Figure 3 shows the schematic of the measurement sequences.

## Results and Discussion

### Measurements of the re-amorphization of a single wire

**Figure 4:**
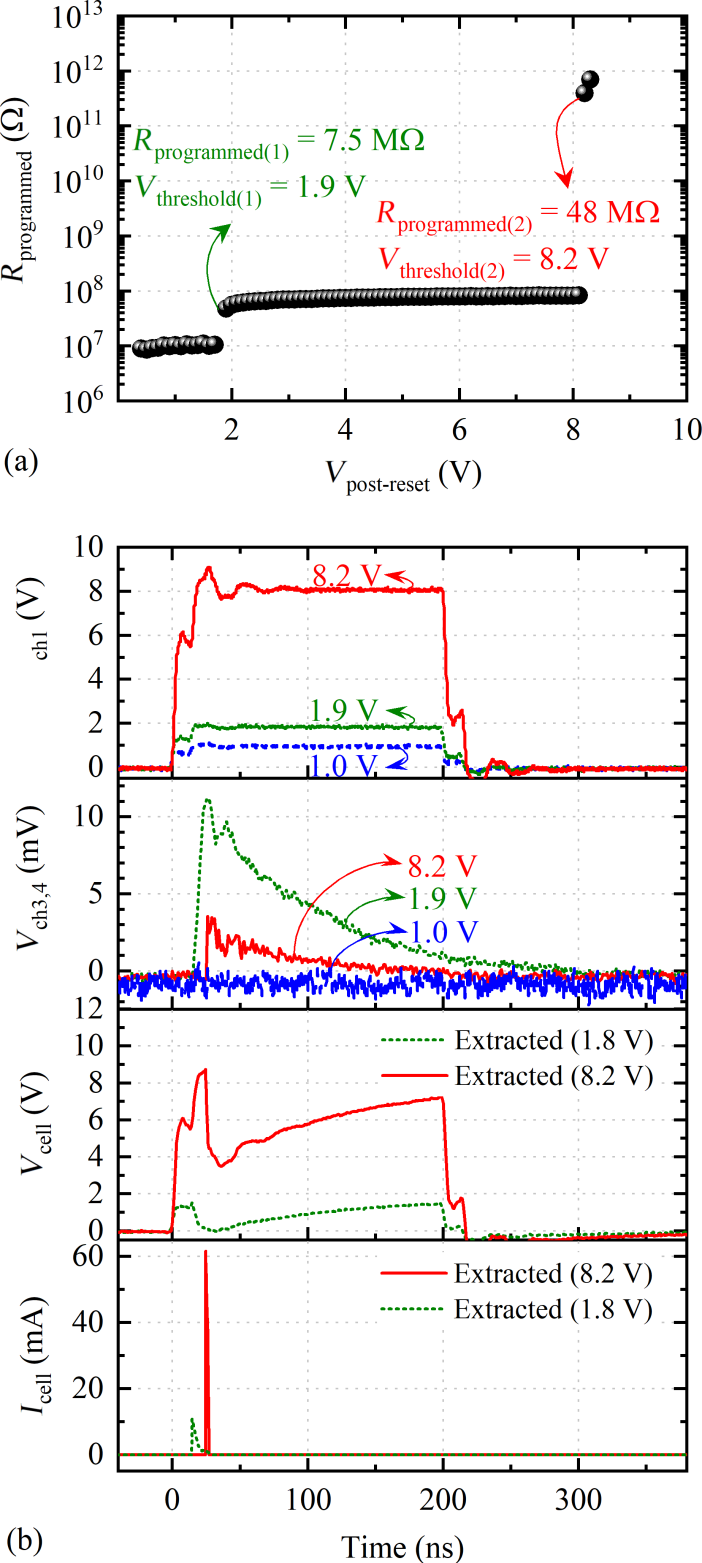
Repeated post-reset pulses of 200 ns duration and 0.4 to 10.0 V amplitude (with an increment of 0.1 V) applied to an amorphous cell with *L*_GST_ ≈ 470 nm, *W*_GST_ ≈ 130 nm, and *t*_GST_ ≈ 50 nm, initially programmed to approx. 7.5 MΩ. (a) Evolution of *R*_programmed_ as a function of the post-reset pulse amplitude (*V*_post-reset_). (b) 1st and 2nd rows: example waveforms of applied voltage at channel 1 (*V*_ch1_) and measured voltage at channel 3,4 (*V*_ch3,4_) during an unsuccessful re-amorphization (blue dashed line), a successful re-amorphization at *V*_post-reset_ of 1.9 V (green dotted line), and a breaking episode when electrical connections are lost at *V*_post-reset_ of 8.2 V (red solid line). 3rd and 4th rows: These voltage waveforms for *V*_post-reset_ of 1.9 and 8.2 V are input in the SPICE circuit simulations and the resulting cell voltage (*V*_cell_) and current (*I*_cell_) are extracted by modeling the experimental setup circuitry.

**Figure 5:**
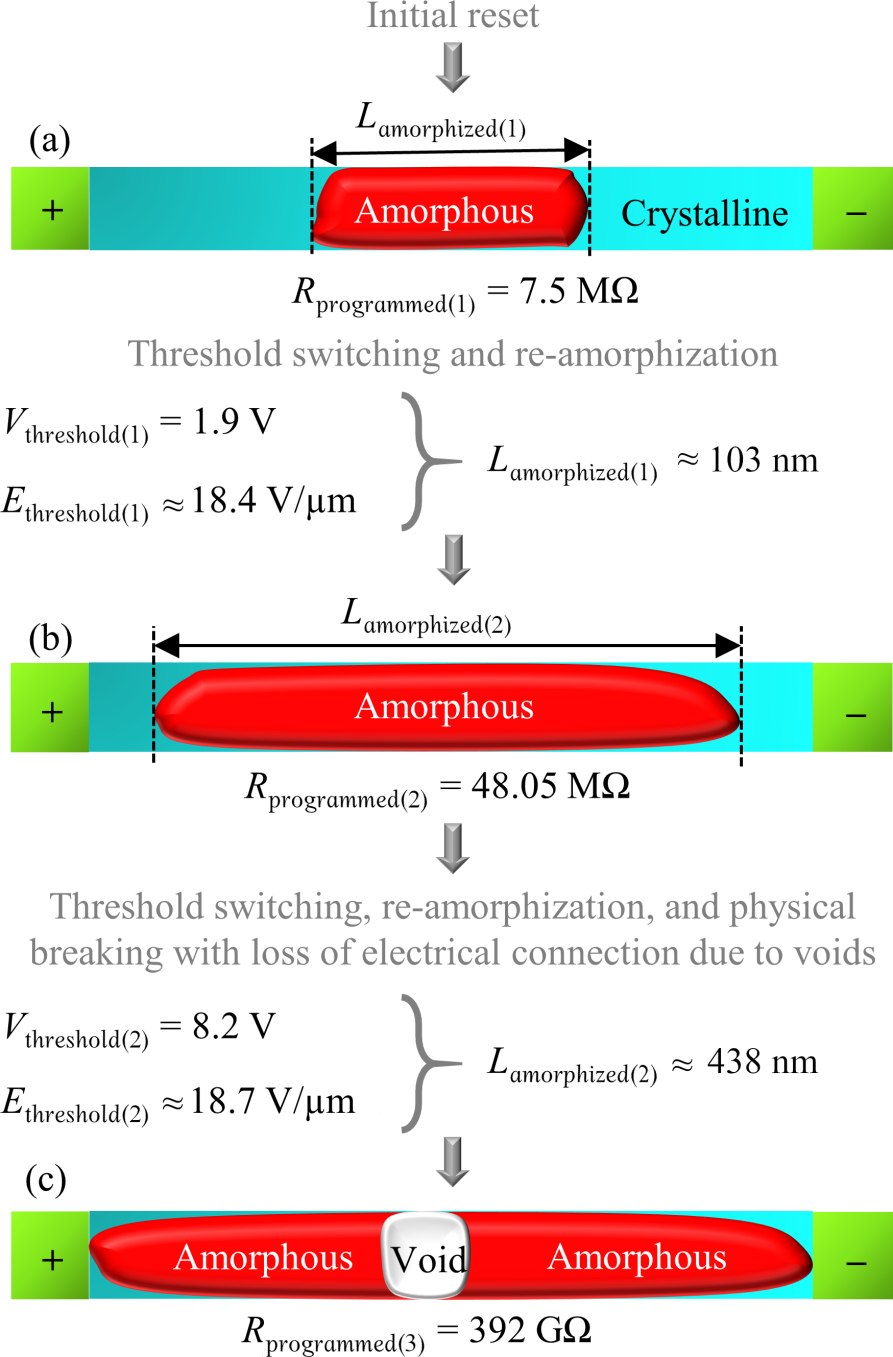
Schematic illustration of the calculation of the amorphized length for two post-reset re-amorphization episodes shown in Figure 4: (a) after initial reset, (b) after first re-amorphization at *V*_threshold(1)_ of 1.9 V, (c) after a second re-amorphization and physical breaking with loss of electrical connection at *V*_threshold(2)_ of 8.2 V. Threshold voltage values indicate the voltage at which the already existing amorphous region experienced electrical breakdown during threshold switching.

An example of the re-amorphization procedure on an initially amorphized cell is shown in Figure 4a, with *R*_programmed_ plotted as a function of the applied voltage at channel 1, *V*_ch1_ = *V*_post-reset_. This cell was initially amorphized at 7.5 MΩ, with a certain amorphized length, *L*_amorphized(1)_. A schematic of the possible phase distribution in the cell after the initial reset is shown in Figure 5a. The amorphous cell started to show the usual upward resistance drift [25], which was unaffected by the early lower *V*_post-reset_ pulses. After a *V*_post-reset_ of 1.9 V was applied, *R*_programmed_ drastically increased from the drifted amorphous resistance value of 10.55 MΩ to 48.05 MΩ (Figure 4a). The measured voltage at the channel 3 and 4 termination resistors, *V*_ch3,4_ showed a sudden overshoot (green dotted line, Figure 4b, 2nd row). This *V*_ch3,4_ amplitude was significantly higher than the barely noticeable *V*_ch3,4_ magnitude for all lower *V*_post-reset_ amplitudes. An example of *V*_ch3,4_ for *V*_post-reset_ of 1 V is plotted in Figure 4b to show the difference (2nd row, blue dashed line). Even though we monitored all waveforms, we only recorded a few to avoid additional time to elapse between the amorphization and threshold switching.

The pulse amplitude of 1.9 V caused a threshold switching, by means of an electrical breakdown of the previously existing insulating amorphous region of length *L*_amorphized(1)_. Therefore, 1.9 V is the threshold voltage, *V*_threshold(1)_, for the breakdown of length *L*_amorphized(1)_. The increased amorphous resistance indicates a larger effective length of the new amorphous region, *L*_amorphized(2)_ (Figure 5b), even though any voids formed would also alter the overall cell resistance. The cell resistance, again, started to drift upward and stayed unaffected by the next few post-reset pulses, until the next threshold switching event at *V*_threshold(2)_ of 8.2 V of the amorphized region of length *L*_amorphized(2)_. At this point, the wire itself physically broke and the electrical connection was lost, possibly due to void formation in the middle of the cell (Figure 5c).

### Cell voltage and current extraction obtained via circuit simulation

**Figure 6:**
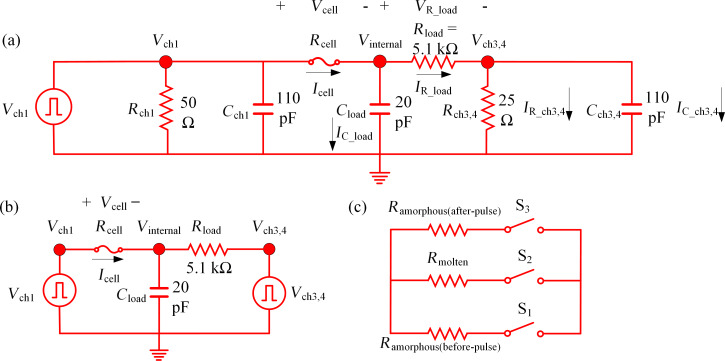
(a) Approximate circuit model of an experimental setup with measured parasitic capacitance and resistance values. (b) Simplified circuit model simulated in SPICE with the measured *V*_ch1_ and *V*_ch3,4_ waveforms. (c) Modeling of GST resistance switching with three switches in SPICE.

A SPICE simulation was performed to extract the cell voltage and current (*V*_cell_ and *I*_cell_) by inputting *V*_ch1_ and *V*_ch3,4_ as the voltage sources. Figure 6a illustrates the approximate circuit model of the experimental setup (shown in Figure 2b) with the following components: channel 1 with a termination resistance of *R*_ch1_ = 50 Ω, a coaxial cable to channel 1 termination with a capacitance of *C*_ch1_ = 110 pF, a probe arm to the load resistor with a capacitance of *C*_load_ = 20 pF, a load resistance, *R*_load_, of 5.1 kΩ. In addition, the channel 3,4 has a termination resistance in parallel of *R*_ch3,4_ = 50 || 50 Ω = 25 Ω and the capacitance value of the coaxial cable to the combined terminations of the channels 3,4 is *C*_ch3,4_ = 110 pF. The cell resistance values, *R*_cell_, used for the SPICE simulations are listed in Table 2.

**Table 2:** Cell resistance values, *R*_cell_, used for the two re-amorphization events in the simulations.

Re-amorphization events	*R*_amorphous(before-pulse)_ (MΩ)	*R*_molten_ (Ω)	*R*_amorphous(after-pulse)_ (MΩ)

1st re-amorphization (1.9 V)	10.55	140	48.05
2nd re-amorphization (8.2 V)	82.66	140	392656.81

Figure 6b shows the simplified circuit model in which the parasitic capacitances and termination resistances for both channel 1 and channel 3,4 are already taken into account with the measured *V*_ch1_ and *V*_ch3,4_. *R*_cell_ is switched between the measured amorphous resistance before pulse, *R*_amorphous(before-pulse)_, the resistance of the molten state, *R*_molten,_ of 140 Ω, and the measured amorphous resistance after pulse, *R*_amorphous(after-pulse)_, (Figure 6c) for the two re-amorphization events shown in Figure 4. The *R*_molten_ value is assumed to be 140 Ω for all simulations, which is slightly less than the *R*_crystalline_ value, since melting incorporates a drop in the GST resistivity [26]. Table 2 lists these different resistance values used for the circuit simulations.

Melting and re-amorphization of a GST cell is simulated with three switches S_1_, S_2_, and S_3_ that sequentially turn on and off to include the appropriate *R*_cell_ (Figure 6c). For both re-amorphization cases, the melting is assumed to take place when *V*_ch3,4_ starts to rise and the re-amorphization is assumed to take place right after the peak of *V*_ch3,4_. Table 3 lists the melting and re-amorphization instances for the two re-amorphization events used in simulations.

**Table 3:** Melting and re-amorphization instances (i.e., the switching time values used for two re-amorphization events in simulations).

Re-amorphization events	Melting instance (ns)	Re-amorphization instance (ns)

1st re-amorphization (1.9 V)	14	28
2nd re-amorphization (8.2 V)	24	26

At the starting of the simulation, S_1_ is closed while S_2_ and S_3_ are kept open to use *R*_amorphous(before-pulse)_ as the starting *R*_cell_. During melting, *R*_cell_ is switched from *R*_amorphous(before-pulse)_ to *R*_molten_ by opening S_1_ and closing S_2_, with S_3_ open. For re-amorphization, *R*_cell_ is switched from *R*_molten_ to *R*_amorphous(after-pulse)_ by opening S_2_ and closing S_3_, with S_1_ open.

For both re-amorphization events, *V*_cell_ ≈ *V*_ch1_ at the beginning, when *R*_cell_ ≈ *R*_amorphous(before-pulse)_, since *R*_amorphous(before-pulse)_ ≫ *R*_load_ and most of the applied *V*_ch1_ voltage drops across the cell. When the melting occurs, *V*_cell_ decreases and *I*_cell_ increases sharply, since *R*_molten_ ≪ *R*_load_. When the quenching occurs, *V*_cell_ increases slowly again, since *R*_amorphous(after-pulse)_ ≫ *R*_load_, and *I*_cell_ decreases sharply due to the amorphization (Figure 4a, 3rd and 4th rows).

We note that *I*_cell_ for each of the re-amorphization events consists of a very sharp triangular current pulse (of approx. 15 ns duration and approx. 11 mA amplitude for the 1st re-amorphization and approx. 3 ns duration and approx. 62 mA amplitude for the 2nd event) that occurs during the rising edge of the *V*_ch1_. The very sharp rising edges of *I*_cell_ for the two re-amorphization events can be attributed to the additional capacitive current contributions due to the parasitic capacitances present in the system and to the thermal runaway of the amorphous GST, which also lead to a quick rise of the cell temperature and produce enough Joule heating to induce melting of the material [27]. The sharp falling edges of *I*_cell_ for the two re-amorphization events due to the discharge of parasitic capacitance can initiate quenching and block the conduction path resulting in re-amorphization [28].

The relation between the measured voltage values, *V*_ch1_ and *V*_ch3,4_, and the extracted parameters, *V*_cell_ and *I*_cell_, can be found using the Kirchhoff’s laws:

[4] $$V_{\text{cell}}=V_{\text{ch1}}-V_{\text{internal}}=V_{\text{ch1}}-V_{{\text{R}}_{\text{load}}}-V_{\text{ch3,4}},$$

[5] $$I_{\text{cell}}=I_{C_{\text{load}}}+I_{R_{\text{load}}}=I_{C_{\text{load}}}+I_{R_{\text{ch3}\text{,4}}}+I_{C_{\text{ch}3,4}},$$

[6] $$I_{C_{\text{load}}}=C_{\text{load}}\left(\frac{\text{d}V_{C_{\text{load}}}}{\text{d}t}\right),$$

[7] $$I_{C_{\text{ch3}\text{,4}}}=C_{\text{ch3}\text{,4}}\left(\frac{\text{d}V_{C_{\text{ch3}\text{,4}}}}{\text{d}t}\right).$$

### Measurements of the re-amorphization of twenty-five wires

**Figure 7:**
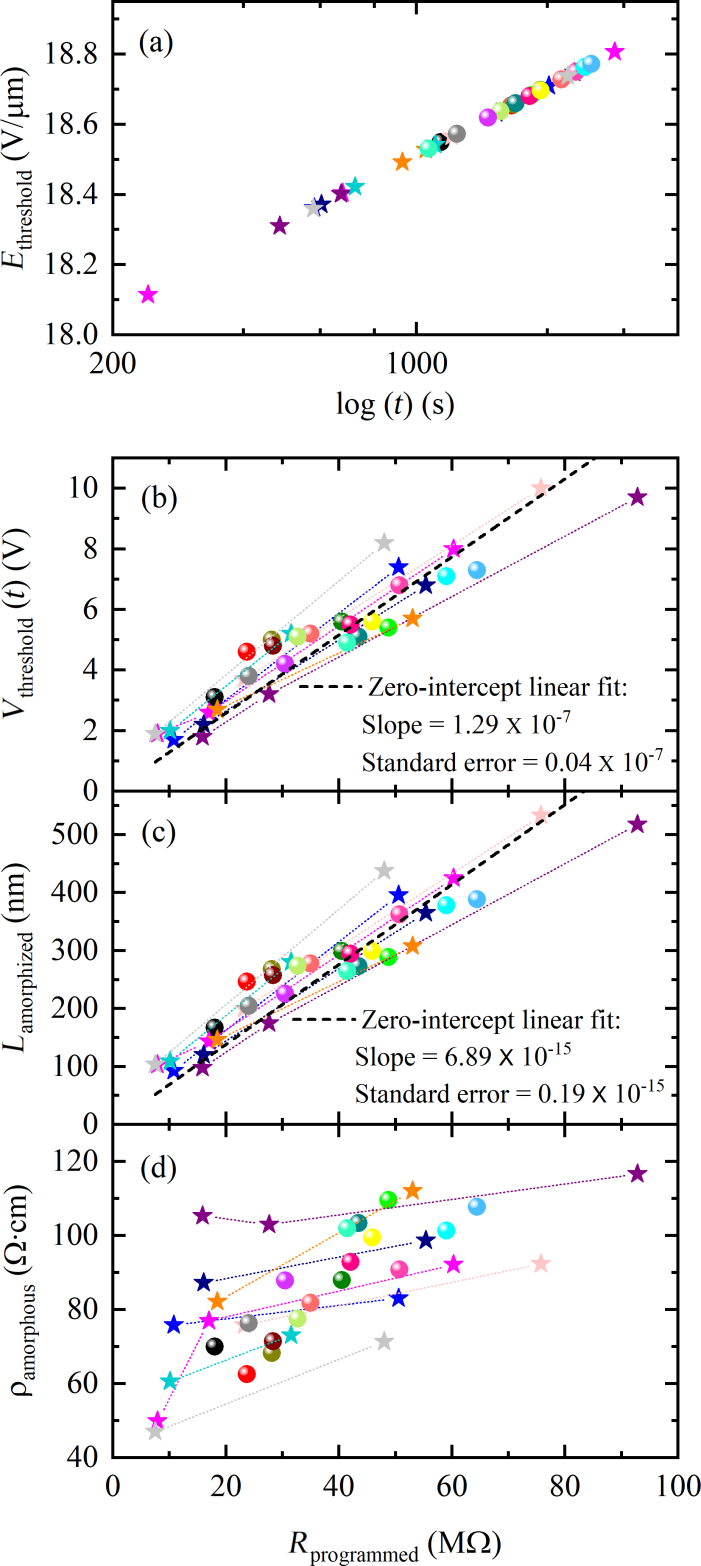
(a) Logarithmic upward drift of the threshold field *E*_threshold_(*t*) for 25 identical cells [20,29] as a function of the elapsed time after amorphization *t*. (b) Measured threshold voltage *V*_threshold_(*t*) as a function of the measured *R*_programmed_. (c) Calculated *L*_amorphized_ values as a function of the measured *R*_programmed_ values, and (d) calculated ρ_amorphous_ (10 s) values as a function of the measured *R*_programmed_ values. The data points for different cells are plotted with different colors. Cells that demonstrated multiple re-amorphization events are plotted with stars connected with dotted lines (8 out of 25 cells) and the ones experiencing a single re-amorphization event are plotted with spheres (17 out of 25 cells).

We repeated the re-amorphization study on 25 cells and measured *V*_threshold_(*t*) values. The extrapolated *E*_threshold_(*t*) (Figure 7a) and the measured *V*_threshold_(*t*) (Figure 7b), both at a certain time *t*, which is different for different measured cells, are used to calculate *L*_amorphized_ (Figure 7c) using Equation 1. Since *L*_amorphized_ remains the same for a given amorphized region, it is only important to consistently extrapolate *E*_threshold_(*t*) at the time *t* when we measure *V*_threshold_(*t*). In Figure 7b, we plotted *V*_threshold_(*t*) as a function of the programmed resistance values measured at 10 s after amorphization (*R*_programmed_ (10 s)). Cells with higher *R*_programmed_ values (17 out of 25 cells) physically broke after the first amorphization (i.e., initial reset) without any intermediate re-amorphization episode and are plotted as spheres in Figure 7b. Cells that experienced one or more re-amorphization episode(s) are plotted as stars connected with dotted lines in Figure 7b. We observed a linear relation between the measured *V*_threshold_(*t*) and *R*_programmed_ (Figure 7b), which in turn is linearly related with the calculated *L*_amorphized_ with a moderate degree of variability (Figure 7c).

There was a cell-to-cell variability in the measured *V*_threshold_(*t*) value for a certain *R*_programmed_ value (Figure 7b). This variability is also propagated to the zero-intercept fitted expression for *L*_amorphized_:

[8] $$V_{\text{threshold}}=R_{\text{programmed}}\times \left(1.29\pm 0.04\right)\frac{\text{V}}{10\;\text{M}\Omega },$$

[9] $$L_{\text{amorphized}}=R_{\text{programmed}}\times \left(68.98\pm 1.94\right)\frac{\text{nm}}{10\;\text{M}\Omega }.$$

**Figure 8:**
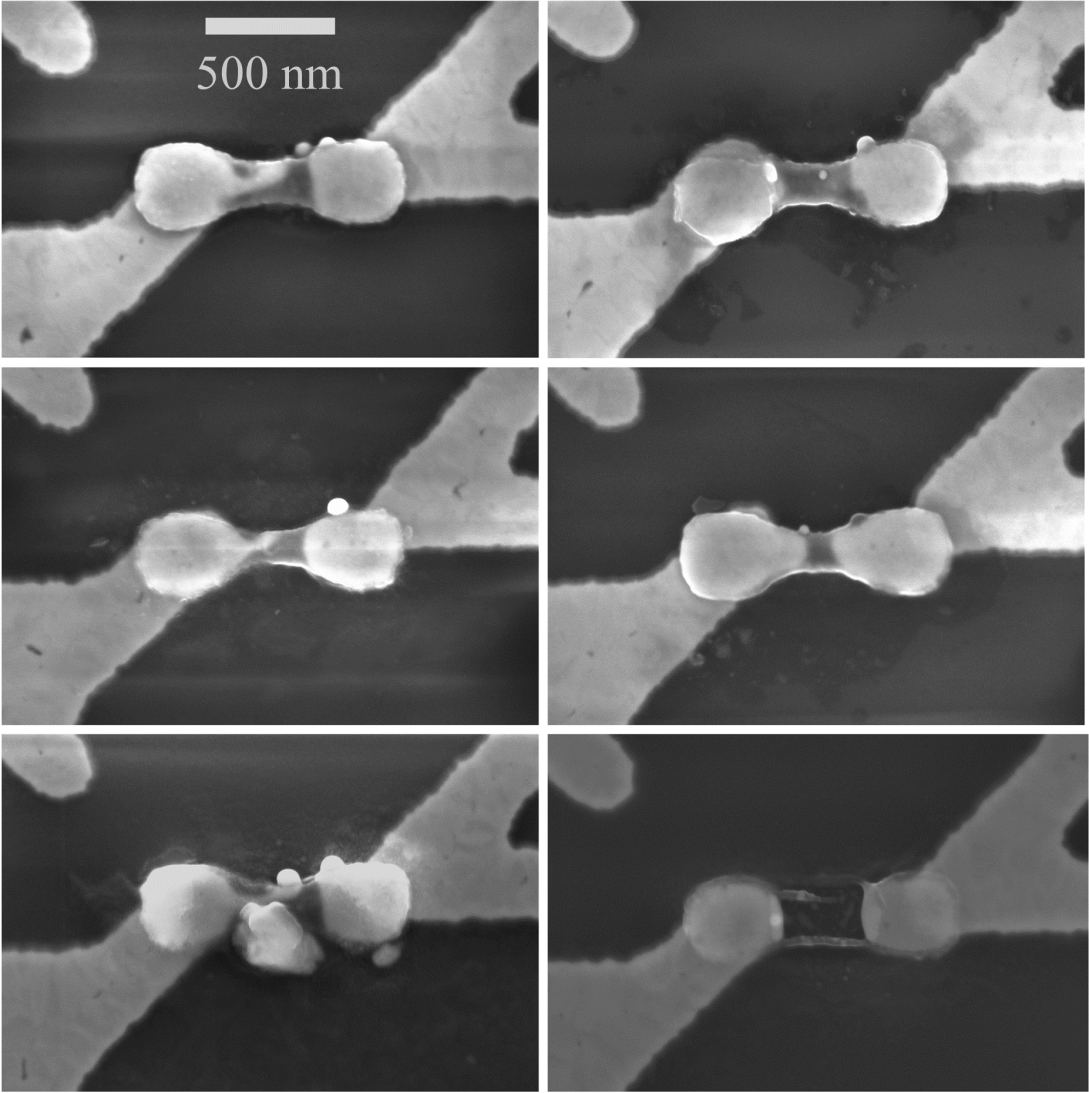
Representative SEM images of six physically broken cells with lost connections, showing significant variability in the void formation despite having similar dimension (*L*_GST_ ≈ 470 nm, *W*_GST_ ≈ 130 nm, and *t*_GST_ ≈ 50 nm) and being programmed to a similar initial *R*_programmed_.

Equation 8 and Equation 9, the zero-intercept linear fits of the data (Figure 7b and Figure 7c), show that for an amorphous region programmed to 10 MΩ, a *V*_threshold_ of 1.29 ± 0.04 V is required for threshold switching, indicating an *L*_amorphized_ of 68.98 ± 1.94 nm. The loss of electrical connection in the wire was confirmed by post-pulse DC *I–V* characteristics and SEM imaging performed after the electrical characterization (Figure 8).

Besides process variations, these variations observed in *V*_threshold_(*t*) and *L*_amorphized_ can be ascribed to variations in the initial *R*_crystalline_ values (shown in Table 1 for the 25 measured cells) due to the random arrangement of the grains in the cells [30], to variations in the shape of the amorphous volumes within the dog-bone-shaped line cells, to slightly different numbers and amplitudes of the applied initial reset pulse(s), which might have amorphized the cell either with a single pulse or with multiple pulses in a more gradual manner, to the random arrangement of any voids created after every reset operation (Figure 8) [31], and to the variable amorphous resistance drift [32] of different cells during the elapsed times between the amorphization and threshold switching.

Understanding the physical origins and detailed properties of these sources of variability in *L*_amorphized_ can contribute to the design of stronger hardware security primitives.

Assuming an ideal uniform cross section of the amorphous regions covering the entire cross-section area *A*_amorphous_ = *W*_GST_ × *t*_GST_ (*W_GST_* ≈ 130 nm, *t*_GST_ ≈ 50 nm), the amorphous resistivity at approx. 10 s after amorphization, ρ_amorphous_ (10 s), can be calculated using the extracted *L*_amorphized_ and the drifted *R*_programmed_ (10 s):

[10] $$\begin{array}{l} \rho_{\text{amorphous}}(10\;\text{s})=\frac{W\times t}{\left(\frac{L_{\text{amorphized}}}{R_{\text{programmed}}(10\;\text{s})}\right)}\\ =\frac{130\times 10^{-9}\text{m}\times \text{50}\times 10^{-9}\text{m}}{(6.90\pm 0.19)\times 10^{-15}\text{m}\cdot \Omega^{-1}}=94.27\pm 2.60\;\Omega \cdot \text{cm}\text{.} \end{array}$$

The calculated ρ_amorphous_ (10 s) is plotted as a function of the *R*_programmed_ values at 10 s in Figure 7d. The calculated ρ_amorphous_ (10 s) of approx. 94 Ω·cm is consistent with our earlier reported value of approx. 148 Ω·cm at approx. 60 s after amorphization obtained from line cells [24] and with 100 Ω·cm reported by Jeyasingh et al. [22]. This close match validates the accuracy of the amorphized length estimation method used in this work.

It is important to note that the calculation of ρ_amorphous_ (10 s) uses extracted *L*_amorphized_ values and values of *R*_programmed_ at 10 s measured under low field (−0.1 to +0.1 V DC sweep) after the post-reset pulse was applied. Hence, ρ_amorphous_ is a low-field resistivity calculation. Sharper rise and fall times are expected to reduce the threshold voltage values due to additional parasitic currents originating from the experimental setup. Reduced parasitic capacitance values from the experimental setup would result in applied *V*_ch1_ voltage pulses with faster and smoother rising and falling edges and enable the *V*_cell_ and *I*_cell_ to closely resemble the applied *V*_ch1_ and the *I*_R_load_, respectively (Equations 4–7) with lesser distortion.

## Conclusion

We propose a method to extract the amorphized length in phase-change memory devices based on electrical measurements. We utilized this procedure to study the variability in amorphized length values in 25 amorphized GST line cells of identical dimensions. Each cell was initially programmed to a similar amorphous resistance level (8–65 MΩ) and then tested with a sequence of post-reset pulses of gradually increasing amplitude, separated by low-amplitude read DC sweeps. When the post-reset pulse amplitude is sufficient, a given cell undergoes threshold switching, melting and quenching and is re-amorphized, as inferred by the measurement and circuit simulation results. The process is continued until the cell breaks, in order to observe as many re-amorphization events as possible.

Each re-amorphization event is observed as a sudden increase in current during the pulse and a higher resistance after pulse, corresponding to a larger effective amorphized volume. The sharp increase in cell current is attributed to the parasitic capacitive current and thermal runaway in amorphous GST. The re-amorphization to increasingly higher resistance levels is also due to the sharp capacitive discharge current at the end of the post-reset pulse.

Using the measured threshold voltage, and calculating an effective drifted threshold field at that switching time, we extracted the length of the amorphized region that experienced threshold switching, for each re-amorphization event, and related it with its previous resistance level. We observe a generally linear relation, but with a significant spread between the amorphized length and the programmed resistance. The amorphous resistivity calculated using the extracted amorphized length and the drifted programmed resistance is approx. 94 Ω·cm, within the range of previously reported values for melt-quenched GST, validating the accuracy of the amorphized length estimation method used in this paper.

The variability in the calculated amorphized length values, based on the measured threshold voltage values, can be attributed to different physical mechanisms, such as the variable amorphous resistance drift and cycling history, the unique amorphous volumes formed, and different crystalline and amorphous initial conditions. SEM images of physically broken cells captured after the electrical measurements show the randomness in the distribution of voids formed during recurrent reset operations, indicating another important factor for the observed variability. Combining these different physical sources of intrinsic variability in cell-to-cell and cycle-to-cycle operations, PCM devices can offer a promising platform for hardware security primitives, in which numerous origins of variability are well-desired features.
